# Annular Elastic Fibrolytic Giant Cell Granuloma: A Dermoscopic Diagnosis

**DOI:** 10.1111/srt.70067

**Published:** 2024-10-04

**Authors:** Ting Zhang, Ling‐Long Cai

**Affiliations:** ^1^ Clinical Medical School Guizhou Medical University Guiyang China; ^2^ Department of Dermatology The Affiliated Hospital of Guizhou Medical University Guiyang China

Esteemed Editor,

Annular elastolytic giant cell granuloma stands as an uncommon clinical condition, with dermoscopic examinations of it being equally infrequent. In this instance, we aim to present a case to dermoscopy for supplementary diagnosis and treatment, thereby clarifying the process of dermoscopic diagnosis.

A 65‐year‐old male farmer arrived at the dermatology clinic, primarily complaining of itching and rashes on the neck and back of both hands, persisting for 3 months. The patient exhibited no additional symptoms and had no prior exposure or use of oral drugs. During the skin check‐up (Figure 1), multiple needle tips were observed encircling the ears (Figure 1a), neck (Figure 1b), and the rear of both hands (Figure 1c), accompanied by a multitude of red‐brown or off‐white papules in varied ring formations, characterized by notably elevated and slender edges, both hard and sleek surfaces, and either thin or regular central skin. Normalcy was observed in the oral lining and toenails.

**FIGURE 1:**
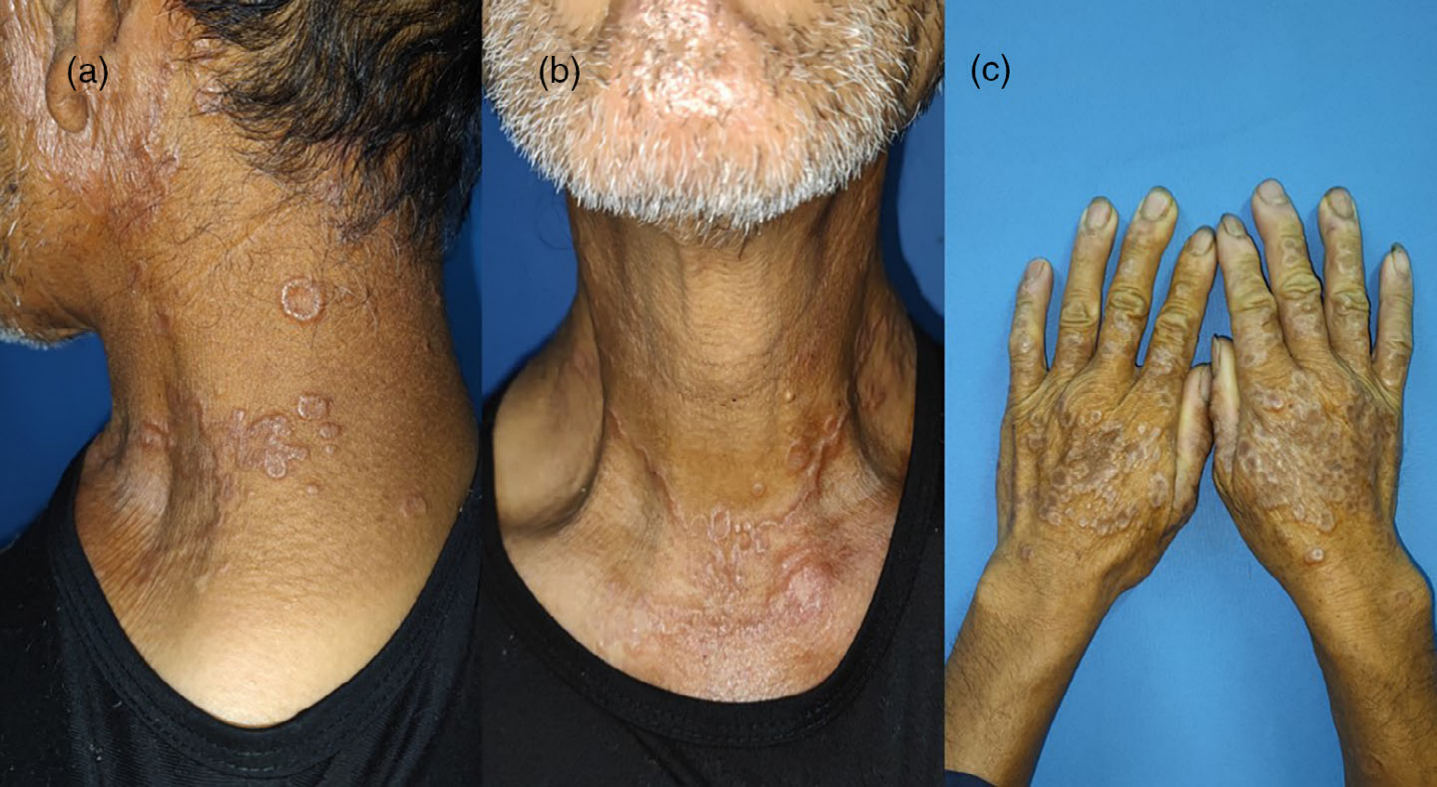
Annular elastic fibrolytic giant cell granuloma: (a–c) clinical.

The assessment utilized dermoscopy, uncovering precisely concentrated, dotted capillaries in round, yellow‐white, disorganized regions against a brown backdrop (Figure 2a,c). The primary histopathological alterations observed were elastic fibrolytic granulomas. The application of EVG staining revealed a notable decrease and phagocytosis of elastic fibers in the lesion zone by macrophages, with the adjacent elastic fibers deteriorating by regular monitoring. (Figure 2b,d). The patients underwent treatment with flumetasone two times a day, followed by regular monitoring.

**FIGURE 2:**
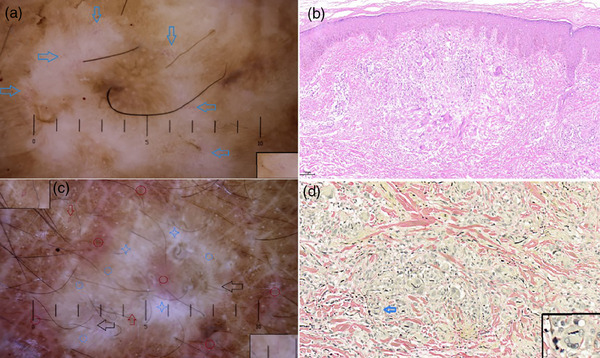
(a) Dermoscopy reveals groupings of dotted capillaries in round, yellow‐white, disorganized spots against a light brown backdrop (indicated by enlarged blue arrows in the box) (HEINE DELTA 20T MINI 3000, 10×, Germany). (c) The Dermoscope displayed a circular, unstructured region against a brown backdrop, a mix of yellow (blue circle) and white (white, four‐pointed star), accompanied by a light erythema (red circle), circular blood vessels (red arrow on the upper left, magnified in the upper left box), and straight blood vessels (red arrow at the bottom, expanded in the lower right box) encircling the center (HEINE DELTA 20T MINI 3000, 10×, Germany). (b) The histopathological analysis revealed a palisade‐resembling invasion of lymphocytes and histiocytes in the dermis, accompanied by multinucleated giant cells (HE,10×). (d) A significant reduction in elastic fibers was observed in the lesion zone, undergoing phagocytosis by macrophages (indicated by blue arrows and enlarged in the box) (EVG, 20×).

Known alternatively as actinic granuloma, annular elastic fibrolytic giant cell granuloma (AEGCG) is a long‐term, non‐malignant skin granulomatous disorder. Typically appearing in middle‐aged adults on areas exposed to light, the rash presents as elevated, ring‐shaped plaques, though it may also appear on areas not exposed [1]. Clinically, the rash is categorized into annular, papular, reticular, or mixed types. As the disease progresses, it can evolve into various types, and a rash is deemed widespread rather than confined if it spreads across multiple regions (like the head, neck, trunk, or any limb) or surpasses 30% of the overall body area [2]. The histopathological examination revealed the presence of elastic fibrolytic granuloma, characterized by macrophages engulfing and vanishing elastic fibers in the lesion zone, whereas those in the adjacent healthy skin deteriorated. Various therapeutic approaches, such as topical, intraleural, or systemic glucocorticoids, chloroquine, hydroxychloroquine, cyclosporine, and phototherapy, have shown differing degrees of effectiveness [3].

This case study marks the inaugural instance of generalized ring‐like AEGCG accompanied by dermoscopic imagery. A comprehensive review of the literature revealed merely three papers detailing the dermoscopic analysis of localized AEGCG. Italian researchers' research identified several dermoscopic characteristics in patients, including (1) an unstructured yellow‐orange region near the rash/active zone; (2) encircling white‐gray scales; three vessels with a consistent reticular focus and center. Regarding the Portuguese case, a dermoscopy showed one widespread erythema and two numerous yellow‐orange dots. Indian patients may observe a 1) a light pink backdrop beneath the dermoscopy; an unstructured yellow‐orange area; (3) a white zone; and (4) pigmentation. Here, it was observed that the patient exhibited a darker complexion, extended duration of illness, and a higher incidence of widespread rashes, leading to certain disordered regions appearing lighter upon dermoscopy. Dermoscopy revealed the presence of erythema and various vascular patterns, predominantly in the white zone of epidermal atrophy. While histopathology is essential for AEGCG, acquiring precise and detailed features of AEGCG through non‐invasive dermoscopy remains crucial to minimize invasive harm. Consequently, we resolved the dermoscopic diagnostic procedure for AEGCG (Figure 3), aspiring for its efficacy in diagnosis, yet further investigation is required to enhance it [4, 5].

**FIGURE 3:**
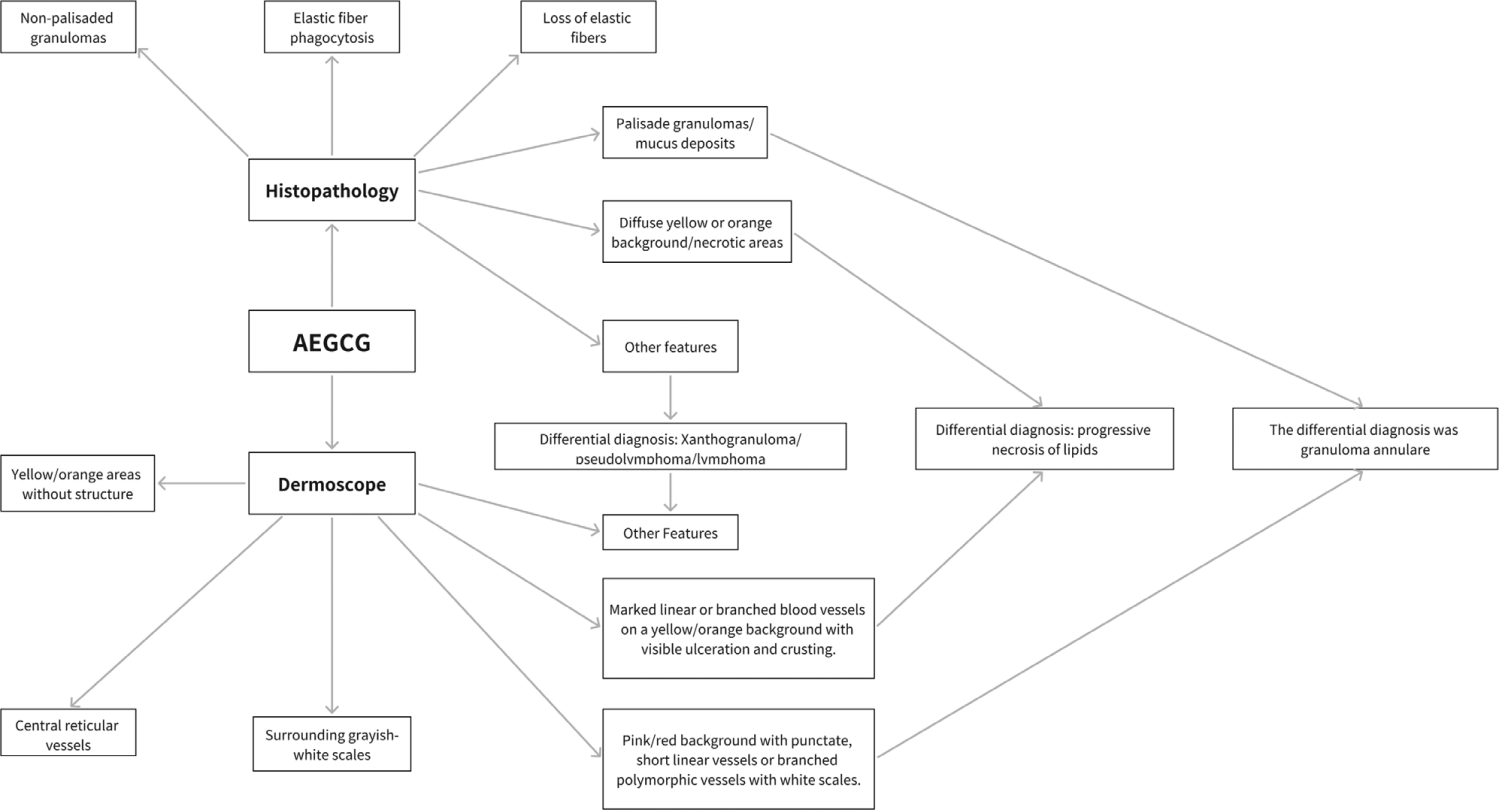
AEGCG process of dermoscopic and pathological diagnosis.

## Conflicts of Interest

Ting Zhang consulted the literature and wrote the manuscript. Ling‐Long Cai discovered the case and revised the manuscript.

## Ethics Statement

Consent for the publication of recognizable patient photographs or other identifiable material was obtained by the authors.

## Data Availability

Data sharing not applicable to this article as no datasets were generated or analyzed during the current study.
